# Glyphosate-Based Herbicides and Their Potential Impact on the Microbiota of Social Bees

**DOI:** 10.3390/toxics13070551

**Published:** 2025-06-29

**Authors:** Juan P. Muñoz, Diego Soto-Jiménez, Anghel Brito, Claudio Quezada-Romegialli

**Affiliations:** 1 Laboratorio de Bioquímica, Departamento de Química, Facultad de Ciencias, Universidad de Tarapacá, Arica 1000007, Chile; 2 Plataforma de Monitoreo Genómico y Ambiental, Departamento de Química, Facultad de Ciencias, Universidad de Tarapacá, Arica 1000007, Chile

**Keywords:** honeybees, bumblebees, microbiota, dysbiosis, glyphosate, glyphosate-based herbicides

## Abstract

Bee pollination is essential for terrestrial ecosystems and crop production. However, the species richness of wild bees and other pollinators has declined over the past 50 years, with some species experiencing dramatic decreases. A key factor in maintaining bee health is their gut microbiota, which plays an essential role in digestion, nutrient absorption, immune function, and resistance to pathogens. Disruptions to this microbiota can severely impact bee health, rendering them more susceptible to diseases and environmental stressors. Glyphosate, one of the most widely used herbicides, has been extensively studied for its effects on various organisms, with increasing evidence indicating its potential to disrupt bee microbiota. This review explores recent research on the effects of glyphosate and its formulations on the gut microbiota of honeybees and bumblebees. It examines species-specific responses, methodological approaches, and broader ecological implications. While evidence indicates that glyphosate can alter the gut microbiome in some bee species, its effects vary depending on exposure conditions, species, and the composition of microbial communities. Additionally, glyphosate formulations containing surfactants may exacerbate these effects. Given the endocrine-disrupting properties of glyphosate, further research is needed to understand the long-term consequences of exposure, especially its impact on hormonal regulation and bee resilience to environmental stressors.

## 1. Introduction

Insect pollination is crucial for both terrestrial ecosystems and agricultural productivity [1]. More than 75% of wild flowering plant species in temperate regions depend on insects for pollination, and nearly 66% of all plant species rely on insect pollinators [2]. Pollinators contribute not only to plant reproduction but also to genetic diversity, ecosystem stability, and food security by facilitating the production of fruits, seeds, and other agricultural commodities [3]. Among these pollinators, bees play a particularly vital role in sustaining biodiversity and enhancing crop yields, with honeybees (*Apis mellifera*), bumblebees (*Bombus* spp.), and numerous wild bee species providing crucial pollination services [4].

Bees can be broadly categorized as either social or solitary species. Social bees, such as honeybees, bumblebees, and stingless bees, live in colonies where individuals share responsibilities such as foraging, brood care, and nest maintenance. These colonies are organized around a reproductive queen and non-reproductive workers [5]. In contrast, solitary bees do not form colonies; each female constructs and provisions her own nest independently [6]. Despite their contrasting lifestyles, both groups are essential pollinators in natural and agricultural ecosystems.

Regardless of their social structure, the foraging behavior of bees exposes them to harmful contaminants. This exposure, along with the alarming decline in bee populations, has raised concerns about a potential “pollination crisis”, in which reduced pollination services could negatively impact agricultural production and ecosystem stability [7,8]. Pollinator declines can lead to reduced crop yields, lower-quality fruits, and economic losses in agricultural sectors that rely heavily on insect pollination [9]. It is estimated that insect pollination contributes more than USD 200 billion annually to global agriculture, underscoring the economic importance of pollinators in food production [10]. Beyond economic consequences, diminished pollination services may disrupt plant–pollinator interactions, leading to cascading effects on entire ecosystems, including declines in plant biodiversity and shifts in community dynamics [11].

Despite extensive research, significant knowledge gaps persist regarding the scale of pollinator declines and their underlying causes. Various environmental stressors have been implicated, including climate change, which affects flowering phenology and pollinator behavior; habitat loss and fragmentation, which reduce nesting and foraging resources; and pathogen and parasite proliferation, such as the spread of *Nosema* spp. and *Varroa destructor*, which significantly impact bee health [12,13]. Additionally, exposure to agrochemicals has been widely recognized as a major threat to pollinator populations [14,15].

Agrochemicals—including insecticides, fungicides, and herbicides—are widely used in modern agriculture to enhance crop yields and control pests, diseases, and weeds [16]. Globally, pesticide use has risen to an estimated 3.70 million tons annually, reflecting a 4% increase since 2021, a 13% rise over the past decade, and a twofold increase since 1990 [17]. However, their prolonged use, even at low doses, along with their environmental persistence, results in accumulation in plants, water, soil, air, and biota, posing significant risks to non-target organisms [18,19,20]. Among the most widely used herbicides, glyphosate has attracted increasing concern due to its potential carcinogenicity and endocrine-disrupting properties [21,22,23]. Emerging evidence suggests that glyphosate-based herbicides (GBHs) may adversely affect learning, memory, reproduction, cell viability, organ function and digestive tract microbiota, across a wide range of non-target organisms, including bee pollinators [24,25,26,27,28].

This review evaluates the impact of glyphosate and its formulations on social bees, with a particular focus on their microbiota. By synthesizing current research, we aim to elucidate the mechanisms through which glyphosate influences microbial communities, its broader implications for bee health, and the factors that modulate bee susceptibility to glyphosate exposure.

## 2. Glyphosate: Chemistry, Usage, and Environmental Impact

Glyphosate ([N-phosphonomethyl]glycine) is a non-selective, systemic, post-emergence herbicide widely employed for controlling broadleaf weeds and grasses in agricultural, urban, and industrial settings [22,29]. Since its introduction in the 1970s by Monsanto under the trade name Roundup, its application has expanded dramatically, particularly following the widespread adoption of genetically modified (GM) crops engineered for glyphosate resistance, such as Roundup Ready soybeans, maize, and cotton [30]. The global use of glyphosate now exceeds 800,000 metric tons annually, with over 200 million hectares of farmland treated each year [29]. In the United States alone, nearly 125,000 metric tons are applied per year, making it the most extensively used herbicide in history [29]. Beyond agriculture, glyphosate is also commonly used for vegetation control along roadsides, railways, public parks, and residential gardens [31]. Due to its high efficacy and low cost, glyphosate has become the dominant herbicide worldwide [32].

The introduction of glyphosate-resistant crops in the mid-1990s revolutionized weed management by allowing post-emergent herbicide application, reducing dependence on mechanical tillage, and promoting no-till farming practices that help mitigate soil erosion and improve carbon sequestration [33]. However, this intensive and repeated use has led to unintended ecological consequences, including the emergence of glyphosate-resistant weed species, shifts in soil microbial communities, and potential risks to non-target organisms. Specifically, glyphosate has been linked to alterations in plant endophytic and rhizosphere microbiomes, as well as disruptions in the gut microbiota of animals residing near agricultural areas [34,35].

Chemically, glyphosate is a polar, organic acid derived from the amino acid glycine [36]. It is highly water-soluble (12 g/L at 25 °C) and strongly adsorbs to soil particles, with its environmental persistence varying based on soil composition, microbial activity, and climatic conditions [37]. Its half-life in soil ranges from a few days to several months, depending on these factors. Microbial degradation serves as the primary pathway for glyphosate breakdown, leading to the formation of aminomethylphosphonic acid (AMPA), its major metabolite [38]. Although AMPA has lower herbicidal activity, it exhibits significant environmental persistence and is frequently detected in soil and water systems, raising concerns about its potential toxicity to non-target organisms.

Commercial formulations of glyphosate are available as isopropylamine, ammonium, or potassium salts, which enhance solubility and plant uptake. In addition, these formulations often contain surfactants, such as polyethylated tallow amine (POEA), a complex mixture of di-ethoxylates of unsaturated and saturated tallow amines to improve penetration into plant tissues and increase herbicidal efficiency [39]. However, certain surfactants, particularly POEA, have been shown to amplify toxicity of glyphosate to non-target organisms, prompting regulatory restrictions in some regions [40].

Glyphosate inhibits the enzyme 5-enolpyruvyl-shikimate-3-phosphate synthase (EPSPS, EC 2.5.1.19) within the shikimate pathway, which is critical for synthesizing aromatic amino acids in plants and certain microorganisms [41,42]. Specifically, EPSPS catalyzes the transfer of an enolpyruvyl group from phosphoenolpyruvate (PEP) to the 5-hydroxy position of shikimate 3-phosphate (S3P), ultimately leading to the production of chorismite [43]. Chorismate is a precursor for essential aromatic amino acids (phenylalanine, tryptophan, and tyrosine) and other metabolites, such as folate cofactors, phenazines, siderophores, and various coenzymes [44,45]. EPSPS enzymes are classified into three groups—Class I, I’, and II—based on biochemical properties and phylogenetic relationships, with Class I and I’ (found in plants and certain bacteria, as well as some archaea for Class I’) being highly sensitive to glyphosate [45,46]. Since the shikimate pathway is present in plants, fungi, bacteria, protozoa, and archaea but absent in animals [47], glyphosate selectively disrupts protein synthesis in plants and microorganisms, ultimately leading to cell death in susceptible organisms [41]. The development of glyphosate-resistant GM crops, which express an EPSPS variant from *Agrobacterium* spp., has enabled these plants to withstand glyphosate exposure and has contributed to the continued expansion of its use [48].

Due to its extensive application, glyphosate residues are frequently detected in soil, water, plant tissues, food products like honey, and even human biofluids [30,49,50]. The presence of glyphosate in honey raises significant concerns about the potential exposure of bees to the herbicide through foraging, further emphasizing the need to study its effects on bee health [19,20,51,52]. Although glyphosate was initially regarded as having a minimal impact on non-target organisms due to its specificity for the shikimate pathway, increasing evidence suggests that it can indirectly disrupt microbial communities across various species. Of particular concern is its potential influence on the gut microbiota of pollinators, especially bees, which play a critical role in both ecosystem health and agriculture [28,53].

## 3. Effects of Glyphosate Exposure on Honeybee Microbiota

In mammals, the gut microbiota is recognized as a dynamic ecosystem shaped by diet, developmental stage, immune system status, stress, antibiotic exposure, and circadian rhythms. These microbial communities play a central role in regulating host metabolism, nutrient absorption, immune homeostasis, and even behavior through gut–brain axis signaling [54]. Disruptions to this balance—commonly referred to as dysbiosis—have been implicated in numerous chronic diseases, including inflammatory bowel disease, obesity, and neurological disorders [55]. The parallels between mammalian and insect microbiome function underscore the ecological and physiological relevance of investigating microbiome disruption across species.

As in the case of mammals, honeybees host a gut microbiome that plays a crucial role in metabolism and immune functions [54,56]. This microbiome includes a diverse array of bacteria, yeasts, and fungi, which interact intricately with the bee host and with each other, supporting developmental processes and defense [57]. The core microbiome of honeybees is composed of five core bacterial taxa: *Lactobacillus*, *Bombilactobacillus*, *Gilliamella*, *Snodgrassella*, and *Bifidobacterium* spp. [58]. Each species provides specific physiological benefits. For example, *Snodgrassella alvi* (*S. alvi*) contributes to immune function and pathogen defense, while *Gilliamella apicola* (*G. apicola*) aids in carbohydrate metabolism and detoxification [56]. Notably, certain bacteria possess the shikimate pathway with Class I or Class II EPSPS enzymes, rendering them hypothetically susceptible to glyphosate. As a consequence, the widespread use of GBHs has raised concerns about their impact on honeybee gut microbiota and overall health [59].

The recent literature has demonstrated that glyphosate exposure disrupts the honeybee gut microbiota in a strain-specific manner [60,61,62]. In this context, the work of Motta et al. has been particularly influential. In 2018, these researchers reported that exposure to glyphosate at a concentration of 5 mg/L, a value falling within environmentally realistic ranges encountered by bees in floral resources, led to a significant reduction in the abundance of key core bacterial taxa, including *Snodgrassella*, *Bifidobacterium* spp., and *Lactobacillus*, while concomitantly increasing the relative abundance of *G. apicola* [60]. Additionally, they found that glyphosate reduces the protective effect of the gut microbiota against opportunistic pathogens and that prolonged exposure exacerbated these effects, resulting in long-term dysbiosis that compromised honeybee health and pathogen resistance [60]. Subsequent studies by the same group in 2020 demonstrated that oral and topical glyphosate exposure under laboratory and field conditions at 169.1 mg/L (1 mM), a concentration representing an upper bound for localized agricultural contamination, significantly altered the composition of the gut microbiota, with *S. alvi* being the most affected species [61]. However, these microbiota alterations did not result in increased mortality under the tested conditions.

In line with the above findings, Blot et al. (2019) reported that glyphosate concentrations between 253.6 and 1268 mg/L, reflecting supra-environmental concentrations, decreased the abundance of *S. alvi* and partially affected *G. apicola*, while concurrently increasing *Lactobacillus* spp. [63]. In the same study, AMPA was also tested and found to have no significant impact on gut bacterial composition in vivo, though some inhibition of *G. apicola* was noted in vitro. Similarly, Castelli et al. (2021) observed a decline in *S. alvi* alongside increases in *G. apicola*, *Lactobacillus kimbladii* (Firm-5), *Staphylococcus* sp., and Enterobacteriaceae at 10 mg/L glyphosate, a field-relevant dose [64]. These studies consistently highlight the vulnerability of *S. alvi* to glyphosate exposure, which has important implications for honeybee health due to its role in gut stability and immune defense [65,66]. Further discussion of its biofilm formation and immunomodulatory functions is provided in Section 5.1.

In a similar vein, a recent study by Motta et al. (2024) investigated the effects of glyphosate on biofilm formation by core honeybee gut bacteria, emphasizing its role in microbial colonization and resilience [67]. The study demonstrated that *S. alvi*, *Gilliamella*, *Bifidobacterium* spp., and *Bombilactobacillus* can individually colonize the bee gut and form biofilms in vitro, which are essential for their persistence [67]. However, glyphosate exposure across a wide range of concentrations (1.69–1690.7 mg/L), encompassing both environmentally relevant and elevated laboratory doses, specifically impaired biofilm formation by *S. alvi*, while only moderately reducing its overall growth. This suggests an interference with extracellular matrix production. Further proteomic analysis revealed that glyphosate-exposed *S. alvi* exhibited a reduced expression of Type VI secretion system proteins—key components in biofilm development—thereby highlighting a potential mechanism through which glyphosate disrupts microbial colonization. Additionally, the study found that commercial glyphosate formulations induced more pronounced effects compared to the active ingredient alone, underscoring the importance of assessing herbicides in their formulated state [67]. These findings emphasize the role of biofilm integrity in maintaining gut homeostasis and suggest that glyphosate exposure may compromise the stability of the honeybee gut microbiome through disruption of biofilm formation.

The effects of glyphosate on honey bee larvae have also been investigated [68,69]. Dai et al. (2018) reported that exposure to 20 mg/L of glyphosate, an intentionally high dose selected to reveal sublethal and microbiota-specific disruptions, significantly reduced larval survival and altered the composition of the midgut microbiota, particularly affecting the relative abundances of Proteobacteria, Firmicutes, and Actinobacteria, which was associated with reduced body weight and developmental impairments [68]. Similarly, Vázquez et al. (2023) demonstrated that field-relevant concentrations of glyphosate induced gut dysbiosis during larval development, followed by post-exposure effects such as delayed metamorphosis, and increased mortality in newly emerged bees [69].

In contrast, Almasri et al. (2022) reported that chronic exposure to an environmentally relevant concentration of 0.1 µg/L glyphosate did not significantly alter the composition of core bacterial species or the total bacterial load in honey bees [70]. Notably, the physiological effects of glyphosate were more pronounced in microbiota-depleted individuals compared to those with a fully established gut microbiota. This included increased LDH activity in the head and elevated GST levels in the midgut, reflecting signs of metabolic stress. The significant differences in overall physiological responses between these groups suggest that a robust core gut microbiota plays a critical role in conferring resilience to pesticide-induced stress in honey bees [70].

Taken together, these findings underscore the detrimental effects of glyphosate on honeybee gut microbiota, highlighting the strain-specific sensitivities of core bacterial taxa and the exacerbating role of commercial formulations containing adjuvants. Additionally, shifts in bacterial composition—such as increased *G. apicola* and *Lactobacillus* spp.—indicate that glyphosate exposure alters the balance of microbial communities, potentially disrupting metabolic and immune functions. Overall, the evidence supports the conclusion that glyphosate exposure can destabilize honeybee gut microbiota, with potential health consequences. A summary of these effects is provided in Table 1.

## 4. Effects of Glyphosate Exposure on Bumblebee Microbiota

Bumblebees (*Bombus* spp.) are indispensable pollinators in many ecosystems, substantially contributing to the production of fruits, seeds, and other agricultural commodities [73]. In Europe alone, their pollination services are valued at over EUR 22 billion annually, emphasizing their vital role in maintaining global food security [74]. Unlike honeybees, bumblebees exhibit annual colony cycles with smaller colony sizes and typically nest underground, factors that can influence both their foraging strategies and exposure pathways to pesticides [75]. Their relatively larger body size and ability to forage under cooler temperatures and lower light conditions allow them to exploit floral resources in environments or seasons less accessible to other pollinators [76]. Furthermore, certain species—such as *Bombus terrestris*—are routinely managed for greenhouse pollination of crops like tomatoes and berries, reflecting their high economic importance [77]. Despite these contributions, bumblebees face alarming declines, with 46% of European species in decline and 24% at risk of extinction, underscoring the need for urgent conservation measures [74].

Bumblebees harbor a gut microbiota that shares several core bacterial taxa with honeybees—*Snodgrassella*, *Gilliamella*, *Lactobacillus*, *Bombilactobacillus*, and *Bifidobacterium* spp.—but also includes distinctive genera such as *Schmidhempelia* and *Bombiscardovia*, reflecting their evolutionary history and ecological niches [78]. This microbial community has coevolved with bumblebees, providing defense against parasites and bolstering resilience to environmental stressors, including heavy metals and pollutants [79]. However, the proximity of bumblebees to intensively cultivated landscapes may directly or indirectly expose them to agrochemicals, including GBHs, through contaminated nectar, pollen, or water sources. Despite the recognized importance of bumblebees as pollinators, relatively few studies have explored how glyphosate or GBHs impact their gut microbiota, highlighting a critical gap in current pollinator research.

Certain studies suggest that glyphosate, in both its pure form and in herbicide formulations, can alter the gut microbiota of bumblebees, though the specific microbial responses may differ. For instance, Motta et al. (2023) exposed *Bombus impatiens* to field-relevant glyphosate concentrations (1.69, 16.91, and 169.1 mg/L), spanning environmentally realistic to moderately elevated exposure levels, and observed a reduction in *Snodgrassella* immediately post-exposure. Notably, no significant changes were detected in other core bacteria—*Bifidobacterium* spp., *Bombilactobacillus*, *Lactobacillus*, and *Schmidhempelia*—implying a relatively resilient microbial community [80]. Similarly, Helander et al. (2023) reported that glyphosate treatment at 10 mg/L and 5000 mg/L, covering both field-relevant and artificially high laboratory concentrations, decreased *S. alvi* in *B. terrestris*, while simultaneously increasing *Candidatus Schmidhempelia*, suggesting that glyphosate may selectively affect certain bacterial taxa. Furthermore, co-formulants in glyphosate-based products appear to exacerbate these effects [81]. Cullen et al. (2023) demonstrated that exposure to RoundUp Optima+^®^ at 1, 10, and 100 mg/L, ranging from field-realistic to elevated doses, modified the *Bombus terrestris* gut microbiota, influencing both fungal communities and proteins associated with oxidative stress and metabolism [82].

Despite these findings, conflicting evidence exists. For example, Straw et al. (2023) reported that exposure to 200 µg of glyphosate per bee—a high acute dose intended to simulate a sublethal field event—had no significant impact on the gut microbiota of *Bombus terrestris*, nor did it influence interactions between glyphosate and the gut parasite *Crithidia bombi* [83]. This stands in contrast with honeybee research, where glyphosate-induced dysbiosis has been linked to increased vulnerability to opportunistic pathogens. Differences in exposure duration—particularly chronic versus acute—may account for these inconsistencies. In line with this, Tang et al. (2023) observed that 10 days of sublethal glyphosate exposure (2.5 mg/L), a concentration reflective of possible environmental exposure, produced no marked changes in the relative abundance of core bumblebee gut bacteria. However, significant alterations in the fungal gut community were observed, including a reduction in *Zygosaccharomyces* and an increase in *Cladosporium* and overall fungal diversity [84]. Finally, another recent study by Hotchkiss et al. (2024) examined the shifts in *B. impatiens* queen gut microbiotas before, during, and after overwintering diapause [85]. Using metagenomic analyses, the authors found that while microbial abundance and community composition changed significantly during diapause, core bacterial taxa largely persisted, and metabolic functions remained relatively stable. Moreover, glyphosate exposure did not significantly hinder gut microbiota recovery in post-diapause queens, suggesting that bumblebees may possess mechanisms for maintaining or reestablishing key microbial functions under stressful conditions [85]. These results suggest that bumblebees may exhibit species-specific detoxification mechanisms that mitigate some bacterial disruptions, while still experiencing significant changes in their fungal microbiome (Table 2).

In summary, although both honeybees and bumblebees experience gut microbiome disruptions following glyphosate exposure, the characteristics and extent of these effects differ significantly between genera. In honeybees, glyphosate exposure consistently induces pronounced and often long-lasting dysbiosis, particularly marked by the reduction in key core taxa such as *S. alvi* and *G. apicola*. These disruptions are frequently associated with compromised immune responses, altered gene expression, and increased vulnerability to pathogens. In contrast, bumblebees exhibit greater microbial resilience, with studies reporting either transient reductions in *Snodgrassella* or minimal changes to the overall bacterial community structure. However, GBHs, which include co-formulants, appear to amplify microbial shifts even in bumblebees, notably by altering fungal communities and increasing the abundance of genera such as *Klebsiella*, *Weissella*, and *Candidatus Schmidhempelia*. These findings suggest that while both genera are affected by glyphosate, the specific microbial taxa disrupted, the duration of dysbiosis, and the resulting physiological consequences are distinct and species-dependent. A summary of these effects is provided in Table 2.

## 5. Functional Consequences of Glyphosate-Induced Dysbiosis in Bees

The evidence reviewed above demonstrates that glyphosate, at specific concentrations, can disrupt the delicate balance of the gut microbiota in both honeybees and bumblebees. Since these microbial communities are vital for maintaining homeostasis, their disturbance may trigger a cascade of negative effects on the overall bee health. In particular, this microbial imbalance can impair key physiological processes such as immune defense, development, and nutrition [86]. The following sections evaluate the potential impacts of glyphosate-induced dysbiosis on physiological processes that are essential for colony viability and the maintenance of ecosystem services.

### 5.1. Pathogen Defense and Immune Function

A balanced adult gut microbiota is crucial for immune homeostasis and the protection of bees against pathogens [87]. Certain gut bacteria play a key role in modulating immune responses and preventing harmful microorganisms from colonizing the gut [88,89]. Consequently, glyphosate-induced dysbiosis may disrupt these protective functions, thereby increasing the vulnerability of bees to pathogens. In this regard, several studies have demonstrated that glyphosate exposure reduces the abundance of *S. alvi* [60,61,62,63,64]. This bacterium forms biofilms in the ileum, creating a mechanical barrier that prevents pathogen invasion. These biofilms are vital for colonization resistance, as they inhibit the attachment of opportunistic pathogens such as *Serratia marcescens* [66].

The importance of *S. alvi* was also highlighted by Blot et al. (2019) [63] and Castelli et al. (2021) [64], who reported consistent reductions in its abundance following glyphosate exposure. These reductions have been associated with compromised gut integrity and an increased risk of infection, given that S. alvi plays a crucial role in maintaining immune balance and gut epithelial protection [65,66]

Furthermore, glyphosate-induced dysbiosis can alter immune gene expression in bees. For instance, research indicates that glyphosate exposure increases the expression of lysozyme—an enzyme that hydrolyzes bacterial cell walls—and glucose oxidase, which produces hydrogen peroxide, an important factor in social immunity [64]. Although these upregulated immune responses may serve as compensatory mechanisms, they are energetically costly and may diminish the bees’ capacity to cope with additional stressors. Glyphosate exposure has also been associated with elevated levels of deformed wing virus (DWV) infection [64], suggesting that dysbiosis can impair antiviral defenses and weaken overall immune competence. In addition, glyphosate appears to affect the expression of vitellogenin, a protein that regulates oxidative stress, immune function, and lifespan. Reduced vitellogenin expression is linked to premature foraging behavior, a shortened lifespan, and heightened susceptibility to environmental stressors. Such effects are particularly concerning as they can diminish colony productivity and reduce the number of long-lived winter bees, which are essential for colony overwintering and survival [64].

Interestingly, the impact of glyphosate on pathogen susceptibility may depend on the pathogen type. For example, while glyphosate increases susceptibility to *S. marcescens* [60], it does not appear to enhance susceptibility to *N. ceranae* [63]. This differential effect might be due to the distinct mechanisms of immune evasion employed by these pathogens, as well as their specific interactions with the gut microbiota. Moreover, the observed sensitivity of honeybees to pathogens under laboratory conditions can be influenced by various methodological and stress-related factors.

Chronic exposure to glyphosate and Roundup formulations also may lead to a decrease in core bacterial taxa, notably *S. alvi*, *Bifidobacterium* spp., and *Lactobacillus Firm-5*, which are essential for nutrient absorption and pathogen defense. In their place, opportunistic bacteria such as *Klebsiella* and *Weissella* can proliferate, likely due to reduced competition from beneficial taxa. This shift compromises gut homeostasis and increases infection risk from environmental pathogens, especially in bees exposed to agrochemical treatments from early developmental stages [90]. Interestingly, this opportunistic proliferation often accompanies a loss of microbial network complexity. Multivariate network analyses have shown that glyphosate disrupts co-occurrence patterns between dominant gut taxa, weakening gut microbial ecosystem resilience and amplifying susceptibility to environmental pathogens [69].

In line with these findings, a recent study showed that glyphosate exposure not only affects the gut microbiota but also alters immune response pathways by downregulating the expression of antimicrobial peptides (AMPs) such as apidaecin and defensin-2, and affects melanization, a crucial immune defense mechanism [71]. This disruption of immune functions further contributes to the heightened pathogen susceptibility observed in glyphosate-exposed bees.

Overall, these findings suggest that glyphosate-induced dysbiosis weakens both mechanical and immune defenses in bees, heightening their susceptibility to bacterial and viral pathogens. The energetic costs associated with mounting these immune responses, coupled with increased oxidative stress and inflammation, can contribute to colony decline and reduce the resilience of bee populations in the face of environmental stressors.

### 5.2. Nutritional Deficiencies, Metabolic Dysregulation, and Development

The gut microbiota contributes to the digestion of complex polysaccharides and the production of short-chain fatty acids (SCFAs), which are essential for energy metabolism [91]. Consequently, glyphosate-induced alterations in microbial composition may reduce the efficiency of nutrient assimilation, leading to malnutrition and energy deficits. These metabolic disruptions can impair foraging efficiency, flight endurance, and overall vitality. Importantly, reduced energy availability may also interfere with critical social behaviors such as brood incubation and nest thermoregulation [92]. The current evidence suggest that glyphosate exposure disrupts the relative abundance of key gut bacteria, which play essential roles in nutrient metabolism. For example, the reduction in *S. alvi*, has been linked to impaired sugar fermentation, reducing the availability of energy-rich metabolites such as acetate and lactate, which are essential for bee energy metabolism [60]. Similarly, the decreased abundance of *Bifidobacterium* spp. and *Lactobacillus* species, which contribute to the fermentation of complex carbohydrates, may result in reduced production of SCFAs, further compromising energy acquisition [80].

Sublethal glyphosate exposure can also affect digestive enzyme activity. For instance, exposure to environmentally relevant concentrations of glyphosate has been shown to significantly decrease gut α-amylase activity, an enzyme crucial for breaking down starches into glucose, thereby impairing carbohydrate digestion and energy production [84]. Additionally, glyphosate exposure reduces the activity of gut proteases, enzymes essential for protein digestion, which can lead to amino acid deficiencies that impair growth and development [82].

On the other hand, glyphosate-induced dysbiosis can indirectly affect nutrient acquisition by altering the gut environment, making it less hospitable for beneficial microbes that assist in nutrient absorption. For example, the increased relative abundance of *G. apicola*, observed following glyphosate exposure, may indicate a compensatory response to dysbiosis, but this shift in microbial composition is associated with reduced production of key metabolic by-products that support host nutrition [60]. In bumblebees, glyphosate exposure has been shown to reduce the relative abundance of *Zygosaccharomyces*, a fungal species associated with fat accumulation, potentially impairing lipid metabolism and energy storage [84]. The impact of glyphosate on energy metabolism can have cascading effects on bee behavior and colony productivity. Reduced energy availability may impair thermoregulation, decreasing colony resilience to temperature fluctuations and reducing brood development rates [92]. Furthermore, energy deficits can limit flight endurance and foraging efficiency, reducing the colony’s capacity to collect nectar and pollen, which are essential for both individual bee nutrition and colony growth [83].

In summary, glyphosate-induced disruptions in gut microbiota composition and digestive enzyme activity can impair nutrient assimilation, leading to energy deficits and metabolic dysregulation. These effects compromise key physiological processes such as thermoregulation, flight endurance, and brood development, ultimately reducing colony productivity. Moreover, the role of microbiota in promoting the energy balance and hormonal regulation underscores its critical contribution to overall bee health and colony sustainability. A summary of core gut microbiota and glyphosate-related effects in honeybees and bumblebees is provided in Table 3.

## 6. Discussion

In mammals, the structure and composition of the microbiome are dynamic, shaped by various factors such as diet, stress, immune responses, aging, and antibiotic exposure. These influences also extend to bees, where environmental and physiological conditions play a crucial role in maintaining the microbial balance [56,87].

In this review, we summarize recent studies examining the effects of glyphosate and its formulations on bee microbiota. Overall, the findings reveal a high degree of variability in microbiome responses between honeybees and bumblebees, emphasizing the need for species-specific evaluations. Regulatory frameworks predominantly rely on honeybees as surrogate species for pollinator risk assessments; however, this approach may not fully capture the distinct microbiota dynamics observed across different bee species. For example, while glyphosate consistently disrupts the honeybee gut microbiota, leading to chronic alterations in core bacterial taxa, bumblebees often exhibit greater resilience or rapid microbiota recovery following exposure. This suggests that microbiota-based effects of glyphosate might be overstated if extrapolated directly from honeybees to other pollinators, highlighting the importance of tailored risk assessments for diverse bee taxa. Moreover, studies on solitary bees have shown that they possess distinct gut microbial communities compared to social bees, with lower microbial diversity and different dominant taxa [94]. Unlike social species, solitary bees lack social transmission routes to acquire gut symbionts from nest mates and are instead exposed directly to environmental microbes upon emergence [95]. Although research on glyphosate exposure in these species is limited, such taxonomic differences in microbiome structure further support the need to broaden ecotoxicological evaluations beyond model species like *A. mellifera*.

On the other hand, glyphosate formulations, such as Roundup, appear to have more substantial effects on microbiota diversity and composition than pure glyphosate alone. Co-formulants, particularly surfactants such as POEA present in many commercial formulations like Roundup, are recognized for their intrinsic biological activity and can independently contribute to toxicity [96]. Several studies reviewed here, including those by Cullen et al. (2023) [82] and Helander et al. (2023) [81], demonstrate that formulations not only exacerbate the disruptions in gut microbiota caused by glyphosate alone but also introduce distinct effects, such as stronger oxidative stress responses, proteomic alterations, and greater fungal dysbiosis. Importantly, the addition of surfactants enhances glyphosate penetration into cells, but these agents themselves may disrupt cell membranes and microbial communities even at low concentrations. This suggests that the toxicity profile of GBHs cannot be solely attributed to glyphosate but must be considered as a cumulative effect of the active ingredient and its formulation adjuvants. The chronic and sublethal effects of these formulations remain largely underexplored in field-realistic settings, where bees are likely to encounter cumulative exposure across various floral resources contaminated with herbicide residues [69]. Given these findings, future ecotoxicological assessments should explicitly differentiate between the effects of technical-grade glyphosate and its commercial formulations to avoid underestimating real-world risks to pollinators.

Although the articles reviewed do not explore the molecular mechanisms by which glyphosate induces dysbiosis, the inhibition of the shikimate pathway is considered the primary mechanism through which it disrupts gut bacteria. However, other studies suggest that glyphosate exposure also increases oxidative stress markers and disrupts microbial metabolic pathways in the guts of bumblebees. This oxidative environment may have sublethal effects on microbial viability, destabilizing the gut ecosystem and increasing susceptibility to pathogens [82,84]. Another hypothesis is that glyphosate may alter the gut pH by disrupting the microbial fermentation processes that produce short-chain fatty acids. Such a shift in pH could create an environment that promotes the growth of harmful pathogens while inhibiting beneficial microbes, ultimately leading to an imbalance in the gut microbiota. Additionally, as a chelating agent, glyphosate may induce the chelation of essential cations in the gut, such as calcium, magnesium, and iron [97]. This chelation could impair microbial and host cellular functions, disrupting nutrient availability and further destabilizing the gut ecosystem.

Among the articles reviewed here, some discrepancies between the results were found, particularly concerning glyphosate effects on microbial diversity. Some studies found significant differences, while others did not. These variations could be explained by differences in exposure routes (oral ingestion versus topical application), developmental stages during exposure (larval versus adult bees), types of matrices used (artificial diets versus natural foods), and the use of pure glyphosate versus formulated products containing surfactants and adjuvants. Other factors include the doses tested (often spanning environmentally realistic to supra-environmental concentrations) and the duration of exposure.

A critical evaluation of the studies reviewed reveals important methodological strengths and limitations that may explain some variability in reported effects. Robust designs were noted in studies such as those by Motta et al. (2018, 2020, 2024) [60,61,67] and Castelli et al. (2021) [64], which included appropriate controls, biological replicates, and chronic exposure protocols reflecting more realistic environmental scenarios. These designs strengthen the reliability of their findings on microbiota disruption. However, a common limitation across many studies is the reliance on laboratory conditions, which, while offering controlled environments, may not fully replicate the complex foraging behaviors and stressors experienced by bees in natural settings. For instance, while Vázquez et al. (2023) [69] incorporated both in vitro and in-hive exposures to better mimic natural routes of glyphosate intake, other studies used direct administration in sugar syrup, potentially overestimating exposure intensity. Field studies such as those by Helander et al. (2023) [81] are valuable in addressing this gap but often face challenges in controlling for confounding environmental variables. Additionally, differences in the developmental stage at exposure, hive health, microbial community baseline composition, and formulation components (pure glyphosate vs. GBHs) introduce additional layers of variability that complicate direct comparisons across studies.

It is interesting to note that several studies, not reviewed here, have highlighted that glyphosate induces toxicity in bees, leading to a range of harmful effects, including impacts on survival, growth, metabolism, behavioral, damage to the midgut epithelium, and increased mortality rates [98,99,100,101]. However, most of these studies have not specifically addressed the potential impact of glyphosate on the microbiota, a crucial component of bee health. Given that the gut microbiome plays a vital role in immunity, nutrient absorption, and hormonal regulation, it is plausible that many of the observed effects—including impaired health and survival—may be mediated by disruptions to the microbiome. This suggests that the gut microbiota is not only a target of glyphosate but could also serve as a potential mediator of the herbicide’s toxic effects on bees. Moreover, as glyphosate is known to interact with endocrine systems in other organisms [21,22], and considering the established role of the honeybee gut microbiota in promoting hormonal signaling, its potential to disrupt hormonal regulation in bees warrants further investigation. Future research should, therefore, focus on understanding how glyphosate exposure alters hormonal pathways in bees, especially considering its role as an endocrine disruptor.

## 7. Conclusions

The impact of glyphosate on bee microbiota involves a complex interplay between chemical exposure, microbial susceptibility, and host species-specific factors. Collectively, studies indicate that glyphosate disrupts the delicate balance of microbial communities in both honeybees and bumblebees, leading to dysbiosis that impairs crucial physiological processes, including immune defense, nutrient metabolism, and pathogen resistance. Additionally, glyphosate formulations, such as Roundup, appear to have more substantial effects on microbiota diversity and composition than pure glyphosate alone. These disruptions may contribute to the decline of bee populations, posing a significant threat to pollination services and causing cascading effects on ecosystem stability.

Given the essential role of the gut microbiome in overall bee health, future research must focus on understanding how glyphosate exposure may interfere with hormonal regulation and amplify the vulnerability of bees to environmental stressors. Such investigations are critical to developing strategies that protect bee health and ensure the sustainability of their vital ecosystem services

## Figures and Tables

**Table 1:** Summary of glyphosate and GBH effects on honey bee microbiota, development, and physiology.

Ref.	Compound Type	Concentration Used	Time of Exposure	Route of Exposure	Developmental Stage	Observed Effects
Motta et al. (2022) [71]	Glyphosate standard (≥95% purity)	(i) In vivo assays: 0, 16.91, 169.1, and 1690.7 mg/L. (ii) Ex vivo assays: 0, 16.91, 169.1, 338.1, 676.3, 1183.5, and 1690.7 mg/L.	5 days	(i) Oral (in sucrose syrup) (ii) Ex vivo (hemolymph assays)	Newly emerged adult bees (1–5 days old); hive worker bees	Downregulation of antimicrobial peptide (AMP) genes (e.g., apidaecin, defensin, hymenoptaecin); dysbiosis (e.g., reduced *S. alvi* and *Gilliamella*); inhibition of melanization at ≥2 mM (only ex vivo); increased immune dysregulation.
Vázquez et al. (2023) [69]	Glyphosate (PESTANAL standard, ≥99.2% purity)	In vitro exposure: 0.07 and 2.5 mg/L	0–144 h (chronic) 72–144 h (subchronic)	Direct ingestion of contaminated food	Larval (primarily 3rd–5th instars)	In vitro exposure: Dysbiosis with loss of core gut bacteria and increase in environmental bacteria; delayed larval development; increased teratogenesis and mortality during larval and pupal stages; surviving adults heavier but with reduced post-emergence survival.
		In-hive exposure: 0.102 mg/L	Post-supplementation with syrup	Indirect ingestion via contaminated honey/beebread		In-hive exposure: Early larval mortality likely linked to hygienic behavior; reduced adult survival; milder dysbiosis compared to in vitro exposure; slight developmental delays.
Ma et al. (2024) [72]	Glyphosate standard (≥99.5% purity)	5 mg/L	10 days	Oral (via sucrose solution)	Newly emerged adult worker bees (1 day old)	Reduced sugar consumption (significant on day 10); decreased survival probability; no significant change in gut microbiota composition or diversity; significant downregulation of glucose dehydrogenase, vitellogenin, esterase *FE4*, and *CYP6AQ1* genes.
Motta et al. (2020) [61]	Glyphosate standard (≥95% purity)	169.07 mg/L	5 days	Oral (feeding with syrup)	Newly emerged adult bees	Significant reduction in *S. alvi* abundance; altered gut microbiota composition; no significant mortality compared to control.
	Roundup ProMax (48.7% glyphosate potassium salt)	(i) 169.07 mg/L a.e. (lab). (ii) 540 mg/L a.e. Roundup (field) (iii) 270–16,200 mg/L a.e. (topical)	(i) 5 days (lab) (ii) Weekly for 1 month (field) (iii) Single dose	Oral (syrup or water) and topical (spray exposure)	Newly emerged and adult bees	Stronger reduction in *Snodgrassella*, *Gilliamella*, and *Bifidobacterium* than with pure glyphosate; increased mortality (dose-dependent) after topical exposure; reduced hive return rates; transfer of glyphosate to honey within hive; increased susceptibility to *Serratia marcescens* infection.
Almasri et al. (2022) [70]	Glyphosate standard. Purity not specified (NS)	0.0001 mg/L	5 days	Oral (in sucrose solution)	Newly emerged adult bees	No significant effect on core gut microbiota composition or total bacterial load; altered physiological markers (e.g., increased LDH activity in head, increased GST in midgut in microbiota-depleted bees); no impact on food consumption or survival; effects more pronounced in microbiota-depleted bees, suggesting gut microbiota buffers toxicity.
Castelli et al. (2021) [64]	Glyphosate standard (Sigma-Aldrich, Burlington, MA, USA, purity ≥95%)	10 mg/L	7 to 14 days (chronic exposure)	Oral (in sucrose solution)	Newly emerged adult bees (≤24 h post-emergence)	Altered gut microbiota composition: ↓ *S. alvi*, ↑ *G. apicola*, *Lactobacillus kimbladii*, *Staphylococcus*; increased alpha and beta diversity; increased expression of lysozyme and glucose oxidase (immune response genes); decreased vitellogenin expression (related to longevity and health); increased DWV replication; significantly reduced lifespan (LT_50_ = 13 days vs. 20 days control).
Blot et al. (2019) [63]	Glyphosate standard (Interchim, Montluçon, France, SS-7701, purity ≥95%)	253.6 and 1268 mg/L	15 days (chronic)	Oral (via sugar syrup)	Adult worker bees (interior workers, overwintering and summer)	Significant dose-independent decrease in *S. alvi*; decrease in *G. apicola*; increase in *Lactobacillus* spp., especially *Firm-5* (at 1268 mg/L); no effect on *Bifidobacterium* spp. or *Alphaproteobacteria*; in vitro growth of *S. alvi*, *G. apicola*, *Bifidobacterium* spp. inhibited; no significant effect on survival or food consumption.
	AMPA standard (Sigma-Aldrich, purity ≥95%)	204.1 and 952.2 mg/L	15 days (chronic)	Oral (via sugar syrup)	Adult worker bees (interior workers, summer)	No significant change in gut bacterial composition in vivo. In vitro: inhibited *G. apicola* at higher concentration (5 mM). No effect on *S. alvi*, *Bifidobacterium* spp. or *Lactobacillus* spp.; no impact on survival or food intake.
Motta et al. (2024) [67]	Glyphosate standard. Purity NS	1.69, 16.91, 169.1, 338.1, 676.3, 1014.4, 1352.5, and 1690.7 mg/L	48 h (in vitro assays)	In vitro (in media with bacterial cultures)	Bacterial strains isolated from adult bee guts	Strain-specific, dose-dependent effects on bacterial growth and biofilm formation; inhibited biofilm formation in *S. alvi*, Gilliamella, and others; upregulation of EPSPS and TrpC enzymes; altered proteomic profile in *S. alvi.*
	Roundup ProMax (48.7% glyphosate potassium salt)	1.69, 16.91, 169.1, 338.1, 676.3, 1014.4, 1352.5, and 1690.7 mg/L a.e.	48 h (in vitro assays)	In vitro (in media with bacterial cultures)	Bacterial strains isolated from adult bee guts	More pronounced effects compared to glyphosate alone; low doses often stimulated growth or biofilm formation, while high doses inhibited both; formulation co-factors likely contributed to divergent outcomes; stronger disruption observed in *Snodgrassella* compared to *Gilliamella* and *Lactobacillus*.
Motta and Moran (2020) [62]	Glyphosate standard. Purity NS	1.69, 6.76, 11.83, 16.91, and 169.1 mg/L	15–20 days (chronic exposure)	Oral (in sucrose syrup)	Newly emerged bees (1-day-old) and bees with established microbiota (5-day-old)	Dose-dependent reduction in *S. alvi and Gilliamella;* increase in *Bifidobacterium and Lactobacillus* (Firm-4/5); greater mortality at ≥0.1 mM; effects observed regardless of timing of exposure (early or late microbiota acquisition); gut microbiota disruption consistent across trials; no significant alpha diversity change but altered community structure.
Motta et al. (2018) [60]	Glyphosate standard. Purity NS	5 and 10 mg/L	5 days (oral), plus 3 days post-reintroduction	Oral (in sucrose syrup)	Adult bees with established microbiota and newly emerged bees	Significant reduction in *S. alvi* and other species (e.g., *Bifidobacterium* spp., *Lactobacillus* Firm-4/5); increased relative abundance of *G. apicola*; impaired colonization during early gut development; increased mortality upon infection with *S. marcescens*; glyphosate-sensitive EPSPS class I associated with reduced bacterial growth.
Dai et al. (2018) [68]	Glyphosate standard (Aladdin, Shanghai, China, ≥99.5% purity)	0.8, 4, and 20 mg/L	4 days (D2–D5 post-grafting)	Oral (in artificial larval diet)	Larval stage	Survival: Significant reduction at 4 and 20 mg/L; larval weight: decreased at 0.8 and 4 mg/L; developmental rate: not significantly affected; gut microbiota: at 20 mg/L, significant alteration in midgut bacterial composition (e.g., ↑ Lachnospiraceae, Prevotellaceae, Ruminococcaceae) and reduced beta diversity; specific taxonomic shifts in response to different concentrations.

This table summarizes findings from selected peer-reviewed studies evaluating the effects of glyphosate and GBHs on different developmental stages of honey bees. All concentrations are nominal values based on preparation methods reported in each study. Abbreviations. AMP: antimicrobial peptide; a.e.: acid equivalent; EPSPS: 5-enolpyruvylshikimate-3-phosphate synthase; LDH: lactate dehydrogenase; GST: glutathione S-transferase; LT_50_: median lethal time; NS: not specified. Arrows indicate changes in bacterial abundance: ↓ denotes decreased abundance, and ↑ denotes increased abundance.

**Table 2:** Summary of glyphosate and GBH effects on bumblebee microbiota, development, and physiology.

Ref.	Compound Type	Concentration Used	Time of Exposure	Route of Exposure	Developmental Stage During Exposure	Observed Effects
Tang et al. (2023) [84]	Glyphosate ammonium salt (30% glyphosate acid, purity NS)	2.5 mg/L (sublethal)	10 days	Oral (via sucrose syrup)	Adult workers	Increased activity of superoxide dismutase (SOD) and prophenoloxidase (PPO), suggesting oxidative stress and immune activation; significantly reduced gut α-amylase activity, suggesting impaired digestion and energy metabolism; no significant effect on glutathione-S-transferase (GST), carboxylesterase (CarE), or protease activities; no significant changes in the core gut bacterial community; significantly altered gut fungal community composition: reduced *Zygosaccharomyces*, increased Cladosporium, and increased fungal diversity.
Motta and Moran (2023) [80]	Glyphosate standard (Caisson Laboratories, Smithfield, UT, USA, purity ≥95%)	1.69, 16.91, and 169.1 mg/L	5–7-day post-exposure monitoring	Oral (via sucrose syrup)	Adult workers	Transient reduction in *Snodgrassella* abundance (in 2 of 4 colonies) after exposure, with recovery by day 7 post-exposure; no significant impact on overall bacterial load or survival; no significant effects on bee weight or syrup consumption.
	Roundup ProMax^®^ (48.7% glyphosate potassium salt)	1.69, 16.91, and 169.1 mg/L a.e.	5–7-day post-exposure monitoring	Oral (via sucrose syrup)	Adult workers	Reduced *Snodgrassella* abundance in some colonies, reversible by day 7; significant reduction in survival post-exposure at 1 mM concentration; increased syrup consumption at 1 mM; no major impact on total bacterial abundance or bee weight.
Helander et al. (2023) [81]	Glyphosate (PESTANAL standard, ≥95% purity)	10 and 5000 mg/L (in 60% sucrose solution)	3 and 5 days	Oral (via colony feeding)	Adult workers	Increased gut microbiota diversity in a dose- and time-dependent manner; reduced relative abundance of *Snodgrasella alvi* (EPSPS Class I—sensitive); increased abundance of potentially resistant genera like *Candidatus Schmidhempelia*, *Acinetobacter*, *and Weissella*; no significant mortality effect.
	Roundup Gold (glyphosate isopropylamine salt, 450 g/L a.i.)	10 mg/L a.e. and 5000 mg/L a.e.	3 and 5 days	Oral (via colony feeding)	Adult workers	Decreased microbiota diversity at low dose; high dose did not reduce diversity further; significant increase in mortality at high dose; disruption of gut microbial composition, with reduced *Snodgrasella* and increased *Klebsiella*, *Candidatus Schmidhempelia*, and *Lactobacillus*.
Straw et al. (2023) [83]	Glyphosate standard (Sigma Aldrich, ≥95% purity)	200 µg (acute single oral dose) equivalent to 4 mg/L (assuming ~50 µL ingestion volume)	48 h	Oral (via sucrose solution)	Adult workers	No significant effects on survival, sucrose consumption, weight change, parasite (*Crithidia bombi*) intensity, or gut bacterial microbiome composition; microbiota diversity and relative abundance of major taxa (e.g., *Snodgrassella*, *Gilliamella*, *Lactobacillus*) remained unaffected.
Cullen et al. (2023) [82]	Glyphosate standard (purity NS)	1, 10, and 100 mg/L	5–10 days	Oral (via 40% sucrose solution)	Adult workers	No significant effects on survival, behavior, or sucrose consumption; significant changes in digestive tract proteome, particularly proteins linked to mitochondrial function, oxidative stress regulation, and structural integrity (e.g., collagen, fibrillin); altered abundance of proteins involved in immune response and detoxification; some minor changes in fungal microbiota, but no significant changes in bacterial community composition.
	RoundUp Optima+^®^	1, 10, and 100 mg/L a.e.	5–10 days	Oral (via 40% sucrose solution)	Adult workers	No significant impact on survival, behavior, or food intake; distinct proteomic alterations from the pure glyphosate treatment: more pronounced changes in oxidative phosphorylation, lysosomal proteins, and lipid metabolism; disruption of fungal gut microbiota, especially reduced Candida abundance and increased abundance of *Tomentella*, *Trichoderma*, *Filobasidium*, and *Archaeorhizomyces*; shared effects with glyphosate on structural proteins (collagen, fibrillin), oxidative stress markers, and signaling pathways.

This table summarizes findings from selected peer-reviewed studies evaluating the effects of glyphosate and glyphosate-based herbicides (GBHs) on different developmental stages of bumblebees. All concentrations are nominal values based on preparation methods reported in each study. In the column “concentration”, “A–B” indicates ranges tested within the same experiment; “A and B” indicates independent tests at distinct concentrations. Abbreviations: a.e. = acid equivalent; EPSPS = 5-enolpyruvylshikimate-3-phosphate synthase (target enzyme of glyphosate); SOD = superoxide dismutase; PPO = prophenoloxidase; GST = glutathione S-transferase; CarE = carboxylesterase; NS: not specified.

**Table 3:** Comparative summary of core gut microbiota and glyphosate-related effects in honeybees and bumblebees.

Bee Species	Core Gut Microbiota ^a^	Glyphosate Sensitivity	Observed Microbiome Effects	Other Physiological Effects
*A. mellifera*	*S. alvi*, *G. apicola*, *Lactobacillus Firm-4*, *Lactobacillus Firm-5*, *Bifidobacterium* spp., *Bombilactobacillus* spp.	High; *S. alvi* and *G. apicola* particularly sensitive; altered biofilm formation and abundance	Dysbiosis *(↓ S. alvi*, *G. apicola*), increased alpha and beta diversity; altered immune-related gene expression; fungal dysbiosis (↑ Candida, ↓ Zygosaccharomyces)	Altered expression of AMPs (defensin, apidaecin), ↓ vitellogenin; increased susceptibility to pathogens; reduced longevity; metabolic changes
*B. terrestris*	*S. alvi*, *G. apicola*, *Schmidhempelia* spp., *Lactobacillus* spp., *Bifidobacterium* spp.	Moderate; dose- and strain-dependent effects on microbial diversity and core taxa	Variable shifts in microbial composition; ↓ *S. alvi*, ↑ *Klebsiella*, *Acinetobacter*, and *Weissella*; fungal diversity changes at low doses of GBHs	Proteomic alterations in digestive tissues (collagen, oxidative stress proteins); high doses of GBH increase mortality
*B. impatiens*	*S. alvi*, *G. apicola*, *Lactobacillus* spp., *Bifidobacterium* spp.	Moderate to low; microbiota more resilient with reversible effects post-exposure	Transient reduction in *S. alvi*, minimal effect on total bacterial abundance or community structure	Slight increase in syrup consumption at higher concentrations; reduced survival only after formulation exposure

^a^ Core gut microbiome data for each bee species were adapted from Voulgari-Kokota et al. (2019) [93]. Arrows indicate changes in bacterial abundance: ↓ denotes decreased abundance, and ↑ denotes increased abundance.

## Data Availability

No new data were created or analyzed in this study.

## Abbreviations

The following abbreviations are used in this manuscript:

AMPA	Aminomethylphosphonic acid
AMP	Antimicrobial peptide
GBHs	Glyphosate-based herbicides
GM	Genetically modified
mM	Millimolar
SCFAs	Short-chain fatty acids
*S. alvi*	*Snodgrassella alvi*
POEA	Polyethylated tallow amine
EPSPS	5-enolpyruvylshikimate-3-phosphate synthase
16S rRNA	16S ribosomal RNA
PEP	Phosphoenolpyruvate
